# Endoscopic Resection of Pituitary Macroadenomas Extending Into the Nasal Cavity: A Case Report and Literature Analysis

**DOI:** 10.7759/cureus.68783

**Published:** 2024-09-06

**Authors:** Chandra Veer Singh, Shraddha Jain

**Affiliations:** 1 Otolaryngology - Head and Neck Surgery, Jawaharlal Nehru Medical College, Datta Meghe Institute of Higher Education and Research, Wardha, IND

**Keywords:** endoscopic surgery, nasal mass, nasopharyngeal obstruction, pituitary macroadenoma, pituitary tumor

## Abstract

Pituitary macroadenomas are benign tumors that typically present with symptoms of hormonal imbalance or visual disturbances due to their location and size. However, in rare instances, these tumors can extend beyond the sellar region into the nasal cavity, leading to unusual clinical presentations. This case report describes a 63-year-old woman who presented with progressive nasal obstruction, episodes of dizziness, and occasional headaches. Physical examination revealed a large, firm mass in the nasopharynx. Nasal endoscopy and computed tomography (CT) imaging confirmed the presence of a pituitary macroadenoma measuring 7.3 x 4 x 4.9 cm, extending from the pituitary gland through the sphenoid sinus into the nasal cavity. The tumor did not affect the optic chiasm despite its significant size, as evidenced by normal visual field tests. The patient underwent successful endoscopic transnasal resection of the tumor, a minimally invasive procedure that allowed for complete removal with minimal morbidity. Postoperative recovery was uneventful, and follow-up imaging showed no residual tumor. The patient reported a significant improvement in symptoms, particularly the resolution of nasal obstruction and headaches. Histopathological examination confirmed the diagnosis of a pituitary macroadenoma. This case highlights the rare presentation of pituitary macroadenomas as nasal masses and emphasizes the importance of considering this diagnosis in patients with atypical nasal symptoms. The successful outcome following endoscopic transnasal surgery demonstrates the effectiveness of this approach in managing complex pituitary adenomas with extensive extracranial extension.

## Introduction

Pituitary macroadenomas are benign tumors greater than 1 cm in size, originating from the anterior pituitary gland [1]. They account for approximately 10-15% of all intracranial tumors and can lead to a variety of clinical presentations depending on their size, location, and hormonal activity [2]. Most commonly, these tumors present with symptoms related to hormonal imbalances, such as acromegaly, Cushing's disease, or hyperprolactinemia, or due to the compression of adjacent structures, such as the optic chiasm, leading to visual disturbances [3]. However, in some instances, pituitary macroadenomas can extend beyond the sellar region into the sphenoid sinus and nasal cavity, causing atypical symptoms such as nasal obstruction, epistaxis, or facial pain [4,5].

The extension of pituitary macroadenomas into the nasal cavity is rare and poses diagnostic challenges, as such presentations can mimic other more common conditions like nasopharyngeal carcinoma, inverted papilloma, or sinonasal polyps [6]. This atypical presentation can lead to delays in diagnosis and treatment, mainly if the symptoms are predominantly nasal rather than endocrinological [7]. Imaging studies, particularly magnetic resonance imaging (MRI) and computed tomography (CT) scans, are crucial in diagnosing and characterizing pituitary macroadenomas with extracranial extensions. These imaging modalities help to assess the extent of the tumor, its relationship with surrounding structures, and its potential impact on the optic chiasm, which is vital for planning surgical intervention [8].

Endoscopic transnasal surgery has emerged as the preferred approach for the resection of pituitary macroadenomas with nasal cavity involvement. This minimally invasive technique allows for direct access to the tumor, with reduced morbidity compared to traditional craniotomy approaches [8]. The endoscopic approach is particularly beneficial in cases where the tumor has extended into the sphenoid sinus and nasal cavity, as it facilitates complete resection with minimal disruption to surrounding tissues [9]. This report presents a rare case of a pituitary macroadenoma that extended into the nasal cavity, presenting primarily as a nasal mass. The case highlights the importance of considering pituitary macroadenomas in the differential diagnosis of nasal masses and underscores the efficacy of endoscopic transnasal surgery in managing such cases.

## Case presentation

A 63-year-old woman presented to the clinic with a chief complaint of persistent nasal obstruction, which had been gradually worsening over the past few months. She also reported experiencing episodes of dizziness, a noticeable loss of appetite, and occasional headaches. The patient’s medical history was unremarkable, with no significant past illnesses or surgeries. Despite the severity of her symptoms, she did not seek medical attention until the obstruction began to impact her quality of life significantly.

**Figure 1:**
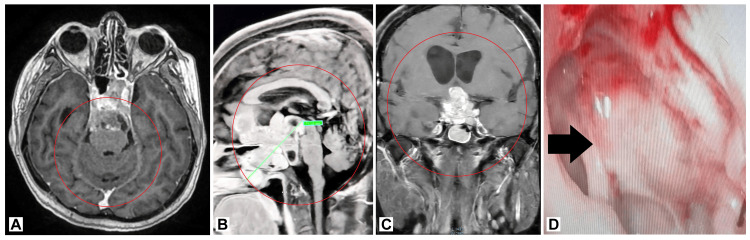
Preoperative imaging of the pituitary macroadenoma. (A) Axial view showing the tumor extending from the pituitary gland into the nasal cavity. (B) Sagittal view illustrating the tumor's extension through the sphenoid sinus. (C) Coronal view highlighting the size and extent of the tumor. (D) Postoperative image showing the successful removal of the tumor with no residual mass.

During the physical examination, a large, firm mass was observed in the nasopharynx, causing partial obstruction of the nasal passage. The mass raised concerns due to its size and location, prompting further investigation. A nasal endoscopy revealed a significant mass occupying the sphenoid sinus and extending into the nasal cavity. A computed tomography (CT) scan of the paranasal sinuses (PNS) and brain was conducted to evaluate the lesion's extent further. The imaging confirmed the presence of a large pituitary macroadenoma measuring 7.3 x 4 x 4.9 cm. The tumor originated from the lower pituitary gland's lower surface, with extensive sella turcica erosion and extension through the sphenoid sinus into the nasal cavity. The tumor had not affected the optic chiasm despite its considerable size, as evidenced by the normal visual field tests (Figure 1).

**Figure 2:**
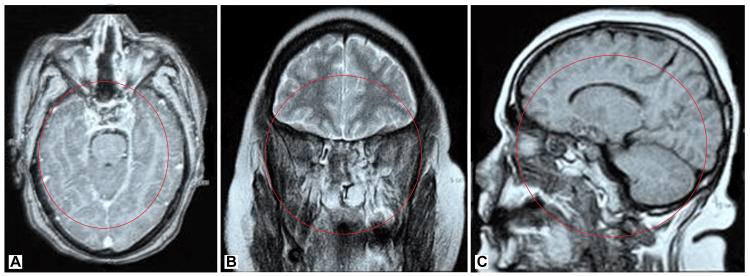
Postoperative day 2 imaging. (A) Axial view of the postoperative site showing no residual tumor. (B) Coronal view confirming the complete resection of the tumor. (C) Sagittal view indicating the clear surgical site following endoscopic resection.

**Figure 3:**
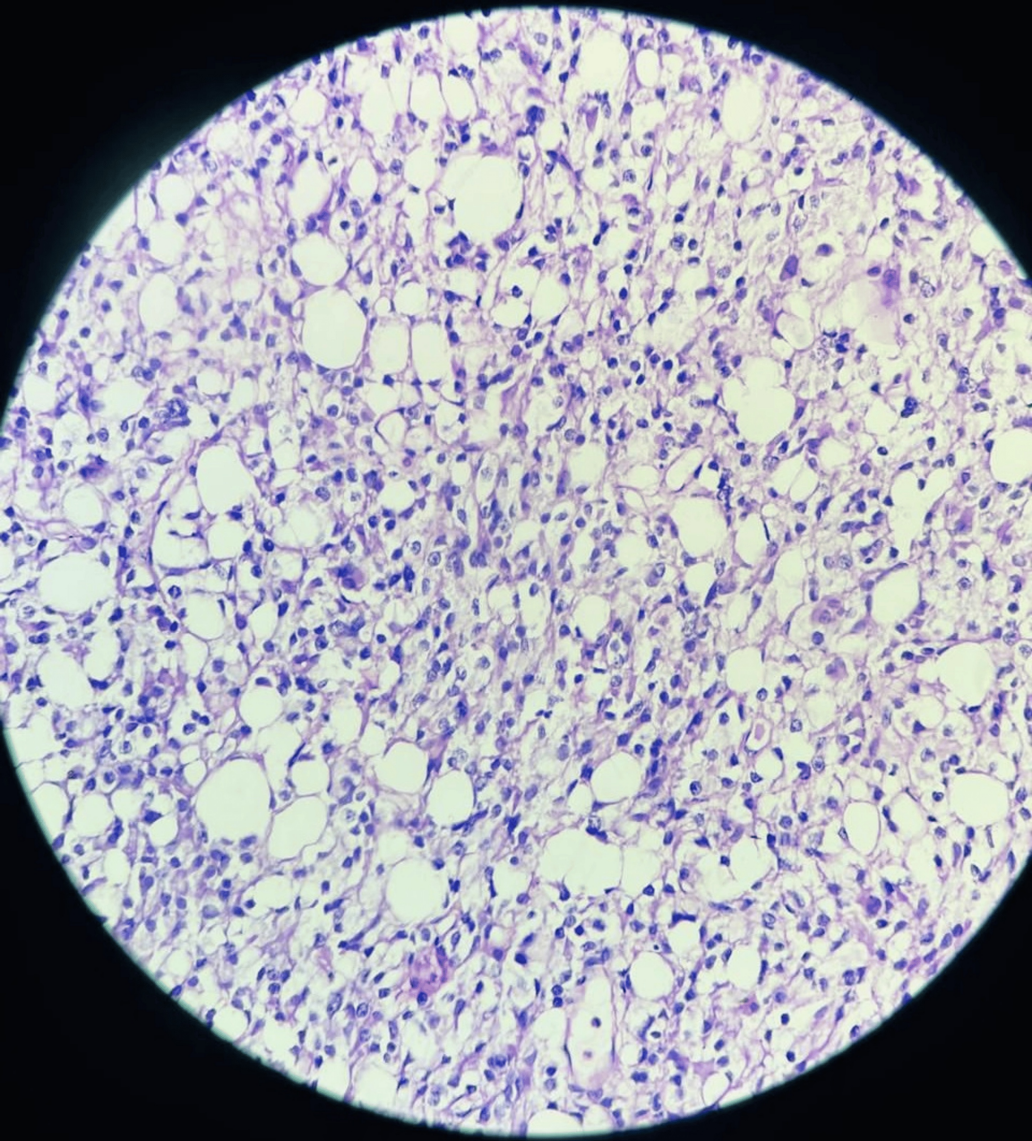
Histopathological examination of the excised tumor, confirming the diagnosis of a pituitary macroadenoma. The analysis reveals typical features of benign adenomatous tissue

Given the tumor’s size, location, and the symptoms presented, a decision was made to proceed with surgical intervention. The patient underwent endoscopic transnasal resection of the tumor, which is a minimally invasive approach that allows for direct access to the tumor with reduced morbidity. The surgery was performed successfully, with the complete removal of the tumor. Intraoperative and postoperative periods were uneventful, with no complications reported. The patient was discharged on the fifth postoperative day and experienced a smooth recovery. Follow-up imaging confirmed no residual tumor, and the patient reported a significant improvement in her symptoms, particularly in the resolution of nasal obstruction and headaches (Figure 2). Histopathological examination of the excised tissue confirmed the diagnosis of a pituitary macroadenoma (Figure 3).

This case highlights the unusual presentation of a pituitary macroadenoma as a nasal mass and underscores the importance of considering such rare diagnoses in patients with atypical nasal symptoms. The successful outcome following endoscopic transnasal surgery demonstrates the effectiveness of this approach in managing complex cases of pituitary adenomas with extensive extracranial extension.

## Discussion

Pituitary macroadenomas are generally known for their classic presentation involving endocrine abnormalities and visual disturbances due to the compression of the optic chiasm. However, their presentation as a nasal mass is uncommon, creating diagnostic challenges for clinicians. The case described in this report illustrates a rare scenario where a pituitary macroadenoma extended beyond the sellar region, eroding the sphenoid sinus and manifesting primarily as a nasal obstruction. The extension of pituitary adenomas into the nasal cavity is rare, with few cases reported in the literature. Such tumors may mimic other more common sinonasal pathologies like inverted papilloma, nasopharyngeal carcinoma, or even chronic sinusitis, often leading to misdiagnosis or delayed diagnosis [10]. In this case, the patient's symptoms of nasal obstruction and headaches, in the absence of endocrine dysfunction or visual impairment, are indicative of the tumor's atypical presentation.

Imaging plays a pivotal role in diagnosing pituitary macroadenomas with nasal extension. MRI is the preferred modality as it provides superior soft-tissue contrast, essential for delineating the extent of the tumor and its relationship with adjacent structures [11]. CT scans are also valuable, especially in assessing bone erosion, as seen in the erosion of the sella turcica in this patient [12]. The absence of optic chiasm involvement in this case was a notable finding, given the large size of the tumor, which typically raises concerns for visual field deficits [13]. The management of pituitary macroadenomas traditionally involves surgical resection, especially in cases where the tumor causes a significant mass effect or extends beyond the sellar region. The endoscopic transnasal approach, as employed in this case, has become the standard of care for such tumors due to its minimally invasive nature, reduced morbidity, and direct access to the tumor through the nasal cavity [14]. This approach not only allows for complete resection of the tumor but also minimizes the risk of complications associated with craniotomy or transcranial approaches [15].

The successful resection of the tumor in this patient, coupled with the absence of postoperative complications, underscores the efficacy of the endoscopic transnasal approach. Postoperative imaging confirmed the complete removal of the tumor, which is crucial in reducing the risk of recurrence [15]. The resolution of the patient’s symptoms, particularly the nasal obstruction and headaches, further highlights the benefits of timely surgical intervention in such atypical cases. The literature on pituitary macroadenomas presenting as nasal masses is limited, with only a handful of case reports available. Most reports emphasize the importance of considering this diagnosis in patients with unusual nasal symptoms, especially when imaging reveals a mass extending into the sphenoid sinus [16]. Moreover, the literature suggests that early diagnosis and intervention are critical in preventing complications such as cerebrospinal fluid leaks, meningitis, or permanent visual loss, which can occur if the tumor is allowed to grow unchecked [17].

## Conclusions

This case report underscores the importance of considering pituitary macroadenomas as a differential diagnosis in patients presenting with atypical nasal symptoms such as persistent nasal obstruction. The unusual presentation of a pituitary macroadenoma as a nasal mass highlights the need for comprehensive diagnostic evaluations, including advanced imaging techniques like CT and MRI, to accurately identify the underlying cause. The successful surgical management of this case through endoscopic transnasal resection demonstrates the efficacy of minimally invasive approaches in treating complex cases where the tumor extends beyond the sellar region. Early diagnosis and timely surgical intervention are crucial in achieving favorable outcomes and preventing potential complications associated with delayed treatment. This case contributes to the growing body of literature on the diverse presentations of pituitary macroadenomas and reinforces the role of endoscopic surgery in managing such challenging cases.
